# Transcriptional Analysis of the Human IgE-Expressing Plasma Cell Differentiation Pathway

**DOI:** 10.3389/fimmu.2019.00402

**Published:** 2019-03-11

**Authors:** Faruk Ramadani, Holly Bowen, Hannah J. Gould, David J. Fear

**Affiliations:** ^1^Randall Centre for Cell and Molecular Biophysics, School of Basic and Medical Biosciences, King's College London, London, United Kingdom; ^2^Asthma UK Centre, Allergic Mechanisms in Asthma, King's College London, London, United Kingdom; ^3^Peter Gorer Department of Immunobiology, School of Immunology & Microbial Sciences, King's College London, London, United Kingdom

**Keywords:** human IgE^+^ B cells, IgE^+^ plasma cell differentiation, gene expression, transcriptomics, apoptosis, cell cycling, allergic disease

## Abstract

IgE is secreted by plasma cells (PCs) and is central to allergic disease. Using an *ex vivo* tonsil B cell culture system, which mimics the Th2 responses *in vivo*, we have recently characterized the development pathway of human IgE-expressing PCs. In this system, as in mice, we reported the predisposition of IgE-expressing B cells to differentiate into PCs. To gain a comprehensive understanding of the molecular events involved in the differentiation of human IgE^+^ B cells into PCs we have used the Illumina HumanHT-12 v4 Expression BeadChip array to analyse the gene expression profile of *ex vivo* generated human IgE^+^ B cells at various stages of their differentiation into PCs. We also compared the transcription profiles of IgE^+^ and IgG1^+^ cells to discover isotype-specific patterns. Comparisons of IgE^+^ and IgG1^+^ cell transcriptional profiles revealed molecular signatures specific for IgE^+^ cells, which diverge from their IgG1^+^ cell counterparts upon differentiation into PCs. At the germinal center (GC) stage of development, unlike in some mouse studies of IgE biology, we observed similar rates of apoptosis and no significant differences in the expression of apoptosis-associated genes between the IgE^+^ and IgG1^+^ B cells. We identified a gene interaction network associated with early growth response 1 (*EGR1*) that, together with the up-regulated IRF4, may account for the predisposition of IgE^+^ B cells to differentiate into PCs. However, despite their swifter rates of PC differentiation, the transcription profile of IgE^+^ PCs is more closely related to IgE^+^ and IgG1^+^ plasmablasts (PBs) than to IgG1^+^ PCs, suggesting that the terminal differentiation of IgE^+^ cells is impeded. We also show that IgE^+^ PCs have increased levels of apoptosis suggesting that the IgE^+^ PCs generated in our *in vitro* tonsil B cell cultures, as in mice, are short-lived. We identified gene regulatory networks as well as cell cycle and apoptosis signatures that may explain the diverging PC differentiation programme of these cells. Overall, our study provides a detailed analysis of the transcriptional pathways underlying the differentiation of human IgE-expressing B cells and points to molecular signatures that regulate IgE^+^ PC differentiation and function.

## Introduction

IgE plays a central role in the pathogenesis of allergic disease (1, 2). Although IgE is the least abundant antibody in the circulation, its binding to the high affinity IgE receptor (FcεRI) on mast cells and basophils is critical for the manifestation of immediate hypersensitivity to allergens and allergic inflammation (1, 2). IgE is secreted by PCs, which represent the terminal stage of B cell differentiation, after immunoglobulin class switching to IgE in precursor B cells (3).

Important advances in understanding the regulation of IgE production have been made over the last decade. The predisposition of IgE-switched cells to develop toward the PC rather than the memory cell lineage is seen in both mouse and human systems (4–10). However, this could not be attributed to differences in the expression levels of the PC differentiation master regulator, Blimp-1 (7, 9). Studies by IgE and IgG1 domain swapping in mouse B cells show that membrane IgE (mIgE) signaling promotes antigen-independent PC differentiation of IgE^+^ B cells (5, 10). The CH2-CH3 extracellular domains and the cytoplasmic tail contribute to this activity, but the key component was the extracellular membrane-proximal domain (EMPD) (5, 10).

The effect of mIgE signaling in PC differentiation has been suggested to involve IRF4 (5, 10), a transcription factor that regulates PC differentiation (11). However, we lack a more comprehensive knowledge of other molecular pathways that likely contribute to this process, especially in humans. Unlike in mouse, two isoforms of mIgE exist in humans, a short form (mIgEs), equivalent to the mouse mIgE, and a long form (mIgE_L_) containing an EMPD that is 52 amino acids longer (12, 13). Expression of the mIgE_L_ by the human IgE^+^ B cells may also influence PC differentiation.

Using an *ex vivo* tonsil B cell culture system, stimulated with IL-4 and anti-CD40 *in vitro* to generate IgE^+^ cells, we have recently characterized the developmental pathway of human IgE^+^ and IgG1^+^ PCs (7). In this system, we demonstrated that there are three discrete stages of IgE^+^ PC development pathway, which we characterized phenotypically as IgE^+^ GC-like B cells (IgE^lo^CD27^−^CD138^−^Bcl6^hi^Pax5^hi^Blimp1^lo^), IgE^+^ PC-like “PBs” (IgE^hi^CD27^++^CD138^−^Bcl6^lo^Pax5^lo^Blimp1^hi^), and IgE^+^ PCs (IgE^hi^CD27^++^CD138^+^Bcl6^lo^Pax5^lo^Blimp1^hi^) (7). A similar IgG1^+^ PC development pathway was also observed. The IgE^+^ cells displayed cell cycle and proliferation rates greater than their IgG1^+^ cell counterparts, and interestingly we also observed that the differentiation of IgE^+^ B cells into PCs is accompanied by the modulation of mIgE_L_ and mIgE_S_ surface expression (7). Here, to better understand the differentiation process of human IgE^+^ B cells into PCs and to identify key regulators of this process, we have used the Illumina HumanHT-12 v4 Expression BeadChip array to define and compare the transcriptomes of *ex vivo* generated IgE^+^ and IgG1^+^ B cells at various stages of their differentiation into PCs.

## Methods

### Cell Cultures

B cells were isolated from the dissected tonsil tissue on a density gradient (GE Healthcare) followed by incubation with aminoethyl isothiouronium bromide-treated sheep red blood cells to rosette T cells (TCS Biosciences). B cells were >95% CD19^+^ as determined by flow cytometric (FACS) analysis. Purified tonsil B cells were induced to undergo class switching to IgE as previously (14). Briefly, 0.5 × 10^6^ freshly purified tonsil B cells were stimulated with IL-4 (200 IU/ml; R&D Europe Systems Ltd.) and anti-CD40 antibody (0.5 μg/ml; G28.5; American Type Culture Collection). After day 7 the population of IgG1^+^ and IgE^+^-switched cells gradually increased to a maximum at 10 days when the cells were harvested for study.

### FACS Sorting of IgE^+^ and IgG1^+^ Cells

Cultured cells were stained with a live/dead fixable stain dye (Life Technologies Ltd.) and anti-CD138 APC (Miltenyi Biotech) followed by fixation with 2% paraformaldehyde. Following washing with RNAsecure (Life Technologies Ltd.) treated PBS, supplemented with 100 U/mL of RNase inhibitor (Bioline Reagents Ltd.) and 5 mM DL-dithiothreitol (Sigma-Aldrich Ltd.), cells were permeabilized with 1% molecular grade triton ×100 (Sigma-Aldrich Ltd.) containing 250 U/mL of RiboSafe RNase inhibitor and 5 mM DL-dithiothreitol and intracellularly stained with anti-IgE FITC (Vector Laboratories) and anti-IgG1 PE (Miltenyi Biotech) for 45 min on ice. The IgE^lo^CD138^−^, IgE^hi^CD138^−^, and IgE^hi^CD138^+^cells and their respective IgG1 counterparts were FACS sorted into melting buffer (Invitrogen) containing 1,600 U/mL RiboSafe RNase inhibitors and 10 mM DL-dithiothreitol and used for total RNA extraction (see below).

### RNA Isolation

Total RNA was isolated using a previously described protocol (7) for the PureLink FFPE total RNA isolation kit (Invitrogen). Briefly, cells were sorted into the melting buffer containing 1600 U/mL RNase inhibitor (Bioline) and 10 mM DTT (Sigma-Aldrich Ltd.) and stored at −80°C before proceeding to the proteinase K treatment for 15 min at 60°C. Subsequently the manufacturers instructions were followed, including the optional DNase digestion. The RNA was further cleaned using the RNeasy Mini Kit RNA Cleanup protocol (Qiagen). RNA concentrations were measured using the NanoDrop 2000 (Thermo Scientific) and RNA integrity assessed using the 2100 Bioanalyser instrument (Agilent Technologies, Inc.).

### Illumina BeadChips Array

cDNA was synthesized and amplified from 40 ng RNA using the Ovation Pico WTA system V2 (NuGEN) and purified using the MiniElute Reaction Cleanup Kit (Qiagen). Yield and purity were measured using the 2100 Bioanalyser instrument and the RNA 6000 Nano kit (Agilent). Four microgram of amplified cDNA was biotin labeled with Encore Biotin Module (NuGen), purified, concentrated and hybridized onto Illumina HumanHT-12 v4 Expression BeadChip array and scanned using the Illumina iScan platform. The data was then subjected to QC analysis and normalization using Illumina's Genome Studio Suite v1.0.

### Microarray and Gene Network Analysis

Assessment of differential gene expression and statistical analysis was performed in Partek Genomics Suite 6.6. Unless otherwise stated 2 way ANNOVA analysis (comparing donor identity and cell phenotype) was undertaken to detect differential expression and the resultant gene lists were obtained by filtering results by FDR < 0.05 and *p* < 0.05 with fold changes >1.5. The PANTHER classification system (15) was used for the gene ontology (GO) analysis of the up-regulated and down-regulated genes. Unsupervised hierarchal clustering was undertaken by K-means clustering of standardized gene intensity values, normalized so that the mean is 0 and the standard deviation is 1 (*z*-score). Finally, gene regulatory networks were investigated using Ingenuity Pathway analysis (IPA) (Qiagen Bioinformatics) to identify known downstream targets of transcription factors (based on Ingenuity knowledge database of mammalian interactions) or using Weighted Gene Co-expression Network Analysis (WGCNA) analysis (16) to identify modules of highly correlated genes. We related these modules to external sample traits using the eigengene network methodology (17).

The array data has been deposited in NCBI's Gene Expression Omnibus (18) and are accessible through GEO Series accession number GSE99948.

### RT-PCR

RT-PCR was performed using TaqMan MGB gene expression assays and TaqMan Universal PCR Master Mix on a Viia7 real-time PCR machine (Applied Biosystems). Gene expression was normalized to an endogenous reference gene 18s rRNA (Hs99999901_s1, Applied Biosystems). Off-the-shelf gene specific qPCR assays were purchased from applied biosystems utilizing Taqman MGB chemistry. All gene specific assays were multiplexed with the 18s endogenous control assay and run in triplicate. SDS software was used to determine relative quantification of the target cDNA according to the 2^−(ΔΔ*ct*)^ method.

### FACS Analysis

To validate some of the differentially expressed genes we fixed, permeabilized, and stained cells as previously described (7). The antibodies used were as follows; anti-IL4R APC (R&D), anti-CD27 FITC (Biolegend), anti-CD38 PE-CY7 (Biolegend), anti-CD20 FITC (Biolegend), anti-IRF4 alexa 647 (Invitrogen), anti-IRF8 APC (Biolegend), anti-BLIMP1 APC (R&D), and anti-active Caspase 3 alexa 647 (BD Biosciences). To determine the rates of apoptosis the IL-4 and anti-CD40 cultured cells were harvested and the dead cells removed using the Easysep dead cell removal kit (Stemcell). The cells were then recultured for 24 h with IL-4 and anti-CD40, followed by staining for Annexin V (eBioscience) and live/dead fixable violet dead stain kit (Life Technologies). Data was collected on a BD FACSCanto (BD Biosciences) and events were analyzed using FlowJo software version 10.4.2 (Tree Star).

## Results

### Transcriptional Profile of GC and PC Associated Genes Along the Differentiation Pathway of IgE^+^ and IgG1^+^ Cells

**Figure 1:**
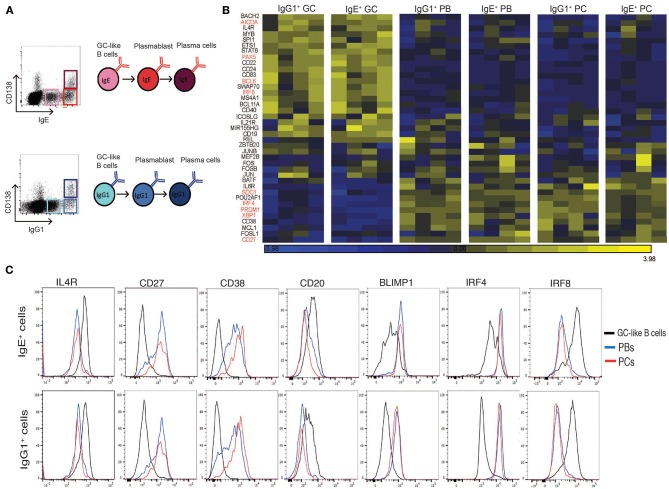
Expression profile of GC and PC associated genes in sorted IgE^+^ and IgG1^+^ cell populations. **(A)** IL-4 and anti-CD40 stimulated tonsil B cells were harvested on day 10 of the culture and surface stained for CD138, intracellular IgE and IgG1 and FACS sorted into GC B cells (IgE^lo^ CD138^−^ and IgG1^lo^ CD138^−^), PBs (IgE^hi^ CD138^−^ and IgG1^hi^ CD138^−^), and PCs (IgE^hi^ CD138^+^ and IgG1^hi^ CD138^+^). **(B)** Heatmap of GC and PC associated genes in each of the sorted IgE^+^ and IgG1^+^ cell populations. Each column represents the gene expression profiles of the different phenotypic cell populations sorted from four different tonsil B cell cultures. **(C)** Flow cytometric validation of seven differentially expressed genes in IgE^+^ and IgG1^+^ cell populations. Data are representative of 6 experiments.

In order to determine the transcriptional profile of IgE^+^ and IgG1^+^ PCs, and their prescursors, after 10 days of culture with IL-4 and anti-CD40, tonsil B cells were sorted by flow cytometry into IgE^+^ and IgG1^+^ GC-like B cells, PC-like PBs and PCs (Figure 1A). Total RNA from the purified cells was isolated reverse transcribed, amplified and biotin labeled prior to transcriptional profiling using the Illumina HumanHT-12 v4 Expression BeadChip array.

To confirm and extend our phenotypic characterization of the IgE^+^ and IgG1^+^ PCs, and their prescursors, we compared the transcriptional profile of known regulators and markers of B cell differentiation into PCs (19–25) (Figure 1B). Genes previously associated with GC reactions were highly expressed in both IgE^+^ and IgG1^+^ GC B cells compared to IgE^+^ and IgG1^+^ PBs and PCs (e.g., *IL-4R* >3-fold, *STAT6* >2-fold, *AICDA* >4-fold, *BCL6* >3-fold). In contrast, genes associated with PC differentiation and functions were highly expressed in both PBs and PCs compared to IgE^+^ and IgG1^+^ GC B cells (e.g., *IRF4* >3.5-fold, *PRDM1* >4-fold, *XBP1* >4-fold). The differential expression of some of the genes was also confirmed at the protein level by flow cytometry (Figure 1C). Overall, the data shows that our previously characterized cell populations displayed a uniform profile with respect to these GC- and PC-associated markers, consistent with the designated phenotype of the populations.

### Distinct Gene Expression Patterns at Different Stages of B Cell Differentiation Into PCs

**Figure 2:**
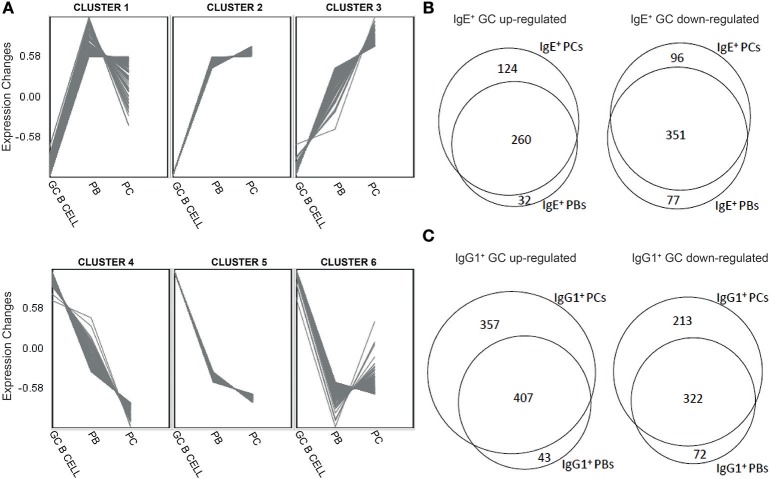
Distinct gene expression patterns and identification of genes unique different stages of B cell differentiation into PCs. **(A)** Clustering of genes differentially expressed along the differentiation pathway of B cells into PCs regardless of the Ig isoform was undertaken by the production of unsupervised Self-organizing Maps (SOM). **(B)** Venn diagrams showing overlaps and differences between genes that were significantly (*p* < 0.05) up-regulated or down-regulated by >1.5-fold in IgE^+^ cells along their differentiation pathway into PCs. **(C)** Venn diagrams showing overlaps and differences between genes that were significantly (*p* < 0.05) up-regulated or down-regulated by >1.5-fold in IgG1^+^ cells along their differentiation pathway into PCs.

To determine the gene expression changes during the differentiation of GC B cells into PCs, irrespective of Ig isotype, we performed a 2 way ANOVA, based on donor identity and cell phenotype, yielding 726 annotated genes that were differentially expressed by >1.5-fold (*P* < 0.05, and FDR < 0.05) between any of the cell types. To identify genes with distinct expression profiles across the three cell types we generated self-organizing maps (SOMs) and identified 6 different patterns of gene expression associated with either negative or positive regulation as cells differentiated into PCs (Figure 2A and Supplementary Table 1).

GO analysis of the clustered genes revealed that cluster 1, identifying genes which peaked at the PB stage, contained genes that were associated with *type I interferon signaling pathway* (GO:0060337, fold enrichment = 27.74), such as *IRF4*, required for PC differentiation (11), and *IRE1-mediated unfolded protein responses* (GO:0036498, fold enrichment = 21.85), which activates *XBP1* (26). Cluster 2 genes, which peaked at the PC stage, are involved in *co-translational protein targeting to membranes* (GO:0006613, fold enrichment = 13.75)*, endoplasmic reticulum to cytosol transport* (GO:1903513, fold enrichment = 59.58), and *endoplasmic reticulum unfolded protein responses* (GO:0030968, fold enrichment = 25.53). Examples include *PRDM1*, the well-known regulator of PC differentiation (27), and *XBP1*, which plays a key role in protein folding, secretion and degradation (28). Expression of genes within cluster 3 also peaked at the PC stage. These genes were involved mainly in *protein N-linked glycosylation via asparagine* (GO:0018279, fold enrichment = 26.99) and *ER-associated ubiquitin-dependent protein catabolic process* (GO:0030433, fold enrichment = 15.64).

In contrast to clusters 1–3, genes within clusters 4–6 were down-regulated as B cells differentiated into PCs. Consistent with the phenotype of cells, these clusters contained genes previously shown to play an important role in establishing, maintaining or mediating GC reactions (19, 20, 24), including *IL4R* (cluster 4), *AICDA, FAS, IRF8* (cluster 5), *BCL6*, and *CIITA* (cluster 6). The main biological processes enriched within cluster 4 are the *cellular response to cytokine* (GO:0034097, fold enrichment = 4.59) and the *regulation of immune responses* (GO:0050776, fold enrichment = 4.02). Genes within cluster 5, primarily restricted to GC cells, were associated with various aspects of cell division, including *DNA unwinding involved in DNA replication* (GO:0006268, fold enrichment = 88.3), *cell cycle phase transition* (GO:0044770, fold enrichment = 7.78) and *DNA replication* (GO:0006260, fold enrichment = 11.88). Genes within cluster 6, repressed particularly in PB cells, are associated with *mitotic cell cycle phase transition* (GO:0044772, fold enrichment = 6.34) and *lymphocyte activation* (GO:0046649, fold enrichment = 4.93).

Since these clusters contain genes with highly correlated expression profiles, we also investigated whether they were known to be regulated by common transcription factors. GO analysis of transcription factor binding sites (TFBS) revealed that all 6 clusters were enriched for certain transcription factor binding sites (TFBS) (Table 1), either specifically enriched in certain clusters (e.g., ETS2 and NFAT in cluster 1; PAX4 in cluster 3; NFY and FOXO4 in cluster 4; E12, PU1, and E2F in cluster 6) or in more than one cluster (e.g., SP1 and LEF1).

**Table 1:** Summary of temporal clusters.

**Cluster**	**Notable genes**	**Top GO biological process (fold enrichment >10, *p* < E-05)**	**TFBS >10 genes**
1	MCL1, IRF4	Type I interferon signaling pathway, endoplasmic reticulum unfolded protein response, cellular response to unfolded protein	ETS2 (11 genes *p* < 0.0012), AP4 (12 genes *p* < 0.0019), SP1 (17 genes *p* < 0.0036), NFAT (12 genes *p* < 0.0088)
2	CD27, PRDM2, IRF1, XBP1	Protein exit from endoplasmic reticulum	SP1 (14 genes *p* < 0.0026), LEF1 (13 genes *p* < 0.0039)
3	CD38, CD79A	Protein N-linked glycosylation via asparagine	SP1 (25 genes *p* < 2.3e-5), LEF1 (21 genes *p* < 0.00026), MYC (11 genes *p* < 0.0006), PAX4 (11 genes *p* < 0.0038)
4	BCL11A, CD19, IL4R	*NS*	MAZ (17 genes *p* < 0.0001), NFY (12 genes *p* < 0.00012), AP4 (12 genes *p* < 0.0005), FOXO4 (13 genes *p* < 0.0011), SP1 (15 genes *p* < 0.0027)
5	AICDA, CCL17, CCL22, FAS, IRF8, MYB	DNA replication	SP1 (17 genes *p* < 0.00026), MAZ (11 genes *p* < 0.0118), LEF1 (12 genes *p* < 0.0152) E12 (11 genes *p* < 0.0153)
6	BATF3, BCL6, CD79B, CD83, SPIB	Mitosis	E2F (11 genes *p* < 7.3e-9), SP1 (33 genes *p* < 7.2e-9), ETS (19 genes *p* < 2.2e07), LEF1 (28 genes *p* < 1.4e-6), E12 (26 genes *p* < 1.5e-6), MYC (15 genes *p* < 1.3e-5), PU1 (12 genes *p* < 1.2e-5)

*The top GO biological processes with the lowest P-value and a fold enrichment threshold of >10 are shown. TFBS significant at 5% threshold and known to regulate >10 genes are shown. For a list of genes related to each of the clusters see Supplementary Table 1. (ns, Not significant)*.

Next, to highlight the transcriptional changes during the PC differentiation of IgE^+^ and IgG1^+^ cells, we constructed a series of Venn analysis diagrams using genes differentially expressed (>1.5-fold change with a *P* < 0.05, FDR < 0.05) along their differentiation pathway into PCs (Figures 2B,C). The comparison showed that both IgE^+^ PBs and IgE^+^ PCs shared a core of differentially up-regulated (351) and down-regulated (260) genes compared to IgE^+^ GC B cells, but also genes that distinguished IgE^+^ PCs (96 up-regulated and 124 down-regulated) from PBs (77 up-regulated and 32 down-regulated) (Figure 2B and Supplementary Table 2). By comparison, while IgG1^+^ PBs and IgG1^+^ PCs also shared a core of differentially up-regulated (322) and down-regulated (407) genes compared to IgG1^+^ GC B cells, the number of differentially expressed genes unique to IgG1^+^ PCs (213 up-regulated and 357 down-regulated) more than doubled in comparison to that of IgE^+^ PCs whereas those of IgG1^+^ PBs were almost unchanged (72 up-regulated and 43 down-regulated) (Figure 2C and Supplementary Table 2). The GO analysis of these genes show that the main biological processes enriched with genes that are either up-regulated or down-regulated in IgE^+^ and IgG1^+^ GC B cells, compared to their more differentiated cell populations, are consistent with their phenotype (Supplementary Table 2).

### The Transcriptional Profiles of IgE^+^ and IgG1^+^ Cells Diverge as PC Differentiation Proceeds

**Figure 3:**
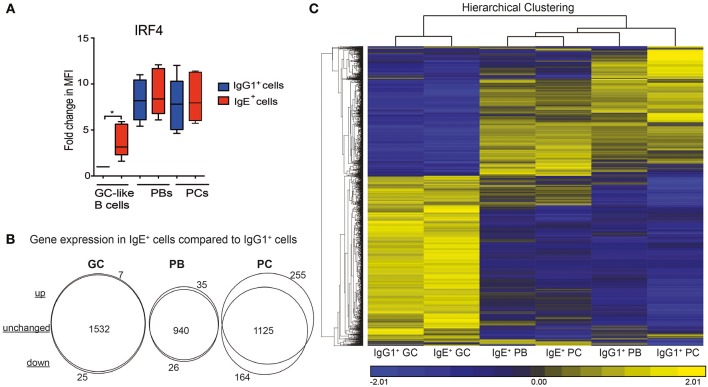
The relationship between the IgE^+^ and IgG1^+^ cells along their differentiation pathway. **(A)** Expression levels of IRF4 in IgE^+^ and IgG1^+^ cells as determine by flow cytometry. Data show the fold change in median fluorescence intensity (MFI) of anti-IRF4 stained cells relative to IgG1^+^ GC-like B cells (*n* = 6). Statistical analysis was performed using the One-Way ANOVA, Dunnett's test (^*^P < 0.05). **(B)** Visualization of gene expression differences between IgE^+^ and IgG1^+^ cells along their PC differentiation pathway. Genes differentially expressed (>1.5-fold, *p* < 0.05) at each IgE^+^ and IgG1^+^ cell differentiation stage underwent a 2-way ANNOVA analysis. The number of genes that were significantly (*p* < 0.05) up-regulated or down-regulated by >1.5-fold in IgE^+^ cells compared to IgG1^+^ cells at GC, PB, and PC are highlighted by the Venn diagrams. **(C)**, Unsupervised K-means hierarchical clustering of all genes differentially expressed in IgE^+^ and IgG1^+^ cells along their differentiation pathway. Each column represents the mean gene expression profile from all four donors of the specified phenotypic group.

We have previously shown that IgE^+^ and IgG1^+^ cells display different biological properties with regards to their differentiation potential (7). Upon examining the expression levels of IRF4, which has been reported to be involved in the PC differentiation of mouse IgE^+^ GC B cells (10), we observed a significantly higher expression of this transcription factor in IgE^+^ cells at the GC stage compared to their IgG1^+^ cell counterparts (Figure 3A). To better understand the molecular pathways underlying these biological differences we carried out a 2-way ANOVA analysis comparing the genes unique to each IgE^+^ and IgG1^+^ cell differentiation stage. As illustrated by the Venn analysis diagrams, IgE^+^ GC B cells share a similar pattern of gene expression with the IgG1^+^ GC B cells (1,532 similarly expressed genes), with only 7 up-regulated and 25 down-regulated genes in IgE^+^ GC B cells (Figure 3B and Supplementary Table 3). At the PB stage of differentiation, IgE^+^ cells had 940 unchanged, 26 down-regulated, and 35 up-regulated genes compared to IgG1^+^ cells. However, at the PC stage, IgE^+^ and IgG^+^ cells diverge in their transcriptional profiles and display a more distinctly different profile with 1125 unchanged, 164 down-regulated and 255 upregulated genes in IgE^+^ PCs compared to IgG1^+^ PCs (Figure 3B and Supplementary Table 3).

To emphasize these diverging transcriptional profiles we subjected genes, the expression of which differed by >1.5-fold across any cell type, to hierarchal clustering (Figure 3C). Clustering confirmed that IgE^+^ and IgG^+^ GC cells were most similar. However, while IgG1^+^ PCs have a very distinct transcriptional profile, IgE^+^ PCs are more closely related to IgE^+^ and IgG1^+^ PBs. This observation was especially surprising, considering that we and others have previously shown that IgE^+^ cells are more prone to differentiation than IgG1^+^ cells (4, 7, 9).

**Figure 4:**
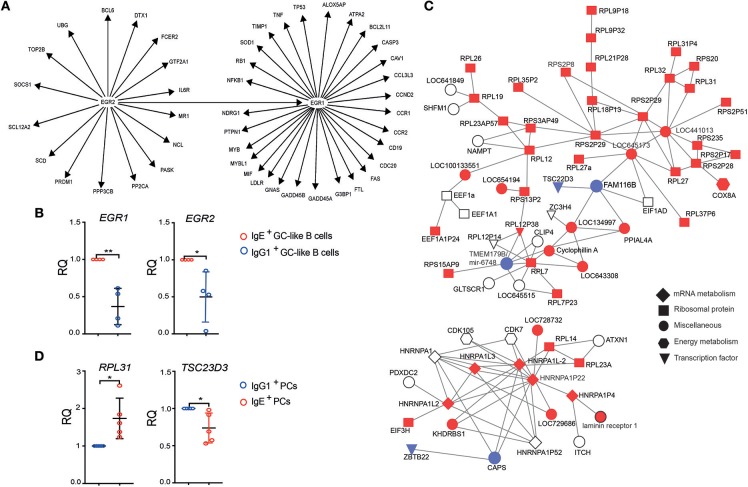
Identification of gene interaction and co-expression networks associated with IgE^+^ PC differentiation. **(A)** IPA was performed on genes that were differentially expressed between IgE^+^ and IgG1^+^ cells (>1.5-fold and *P* < 0.05). The gene network was identified based on the literature contained in the IPA knowledge database. Target genes of the EGR1 and EGR2, shown in the figure, were found to be differentially expressed by more than 1.5-fold (*P* < 0.05) either in IgE^+^ and IgG1^+^ GC-like B cells compared to PBs or PCs or in IgE^+^ cells compared to IgG1^+^ cells along their PC differentiation pathway. **(B)** RT-PCR validation of *EGR1* and *EGR2* expression in IgE^+^ and IgG1^+^ GC-like B cells. Data represent the mean +/– SD of the relative quantification (RQ). Statistical analysis was performed using the *t*-test with Welch's correction (^*^P < 0.05, ^**^P < 0.01). **(C)** Identification of a module of highly correlated genes, by WGCNA analysis encoding a large number of ribosomal proteins, that is enriched in IgE^+^ PCs. In total this network contains 547 genes, however, to improve network visibility only those with a weight above 0.075 are shown. This *de-novo* co-expression network was negatively correlated with the IgG1^+^ PCs (correlation coefficient −0.65, *p* = 0.003). The genes up-regulated (red) or down-regulated (blue) by more than 1.3-fold in IgE^+^ PCs compared to IgG1^+^ PCs, whereas genes with <1.3-fold difference are shown as uncolored. The shape of each node reflects the biological function of each gene, as determined by GO analysis. More detailed information about the top candidate genes displayed in the network can be found in the Supplementary Table 4. **(D)** RT-PCR validation of RPL31, which is up-regulated, and TSC23D3 that is down-regulated in IgE^+^ and IgG1^+^ PCs. Data represent the mean +/– SD of the relative quantification (RQ). Statistical analysis was performed using the unpaired *t*-test with Welch's correction (^*^P < 0.05, ^**^P < 0.01).

To explore the origins of IgE^+^ and IgG1^+^ cell differences, we undertook a gene regulatory network (GRN) analysis using the curated knowledge database in IPA, as well as a data-driven approach using WGCNA (16). IPA analysis on the differentially expressed genes between IgE^+^ and IgG1^+^ GC-like B cells identified a gene interaction network associated with the inducible zinc finger transcription factors, *EGR1* and *EGR2* (Figure 4A). The RT-PCR analysis confirmed the up-regulated *EGR1* and *EGR2* expression in IgE^+^ GC-like B cells (Figure 4B). These transcription factors are known regulators of a number of genes and include those that are down-regulated (*CASP3, MYB, LDLR, GNAS, FTL, CCR2, CCND2*, and *NDRG1*) or up-regulated (*CAV1, FAS, CD19, G3BP1, LOX5AP, NFKB1, MYBL1, TNF, TP53, SOD1*) in IgE^+^ and IgG1^+^ GC B cells compared to their more differentiated cell counterparts. This network also contained genes upregulated (*NCL, FCER2, CDC20, CCL3L3*, and *CCR1*) or down-regulated (*PTPN1, GADD45A, GADD45B, TIMP1, NDRG1*, and *RB1*) in IgE^+^ PCs compared to IgG1^+^ PCs (Figure 4A).

In addition, WGCNA identified a co-expression network, which is enriched in IgE^+^ PCs (*p* = 0.003), containing a large number of ribosomal components and the differentially expressed transcriptional regulator *TCS22D3* and guanine exchange factor (*FAM116B*) (Figures 4C,D and Supplementary Table 4).

Overall these data suggest that the IgE^+^ and IgG1^+^ cells adopt an increasingly different gene expression profile as they differentiate into PCs. The data also provide molecular signatures that may account for some of the differences seen in the later stages of IgE^+^ and IgG1^+^ cell differentiation.

### Proliferative and Apoptotic Associated Genes Differentially Expressed in IgE^+^ and IgG1^+^ Cells

According to the GO analysis, among the most enriched biological processes associated with genes over-expressed in IgE^+^ PCs, compared to IgG1^+^ PCs, were *translation initiation* (GO:0006413, fold enrichment = 12.74), *mitotic cell cycle phase transition* (GO:0044772, fold enrichment = 6.09) and *mitotic cellular division* (GO:0007067, fold enrichment = 4.67), suggesting that IgE^+^ PCs are still cycling (Supplementary Table 3). These observations are consistent with our previously reported data (7), which show that the proliferative and cycling capacity of IgE^+^ PB and PCs is greater than that of their IgG1^+^ cell counterparts.

**Figure 5:**
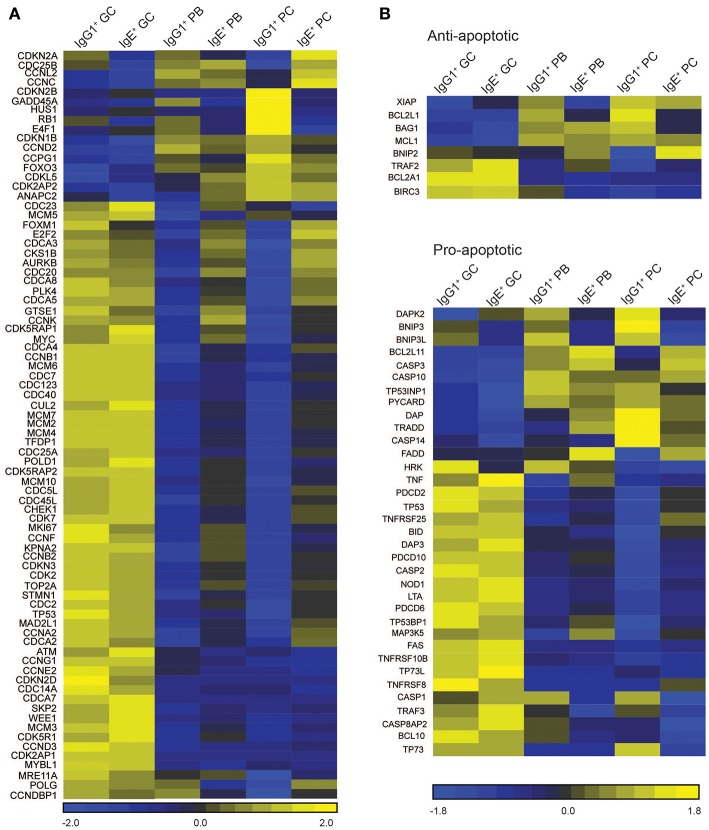
Cell cycle/proliferation-associated genes differentially expressed in IgE^+^ and IgG1^+^ cells. **(A)** Heatmap of cell cycle/proliferation-associated and **(B)** pro- and anti-apoptotic genes differentially expressed along the PC differentiation pathway of both IgE^+^ and IgG1^+^ cells, and differentially expressed in IgE^+^ cells compared to IgG1^+^ cells. Each column in the heat maps shown represents the mean gene expression profile from all four donors of the specified phenotypic group.

There are several differentially expressed genes that correlate with the enhanced proliferation of IgE^+^ cells relative to their IgG1^+^ cell counterparts (Figure 5A). Among these genes, *RB1*, an important regulator of the G1 checkpoint (29), and G*ADD45A*, a regulator of the G2-M checkpoint (30, 31), are upregulated in IgG1^+^ PBs and PCs, but not in IgE^+^ PBs and PCs, when compared to GC B cells (Figure 5A). Other negative regulators of the cell cycle progression up-regulated in IgG1^+^ PBs and PCs include *CDKN2B, HUS1*, and *E4F1*. Conversely, we observe that IgE^+^ PBs and PCs, unlike their IgG1^+^ cell counterparts, up-regulate the expression of a number of genes associated with positive regulation of the cell cycle e.g., *CDC25B, MYC, CSK1B, FOXM1, CDCA3, AURKB, PLK4, CDC20, E2F2* (Figure 5A).

**Figure 6:**
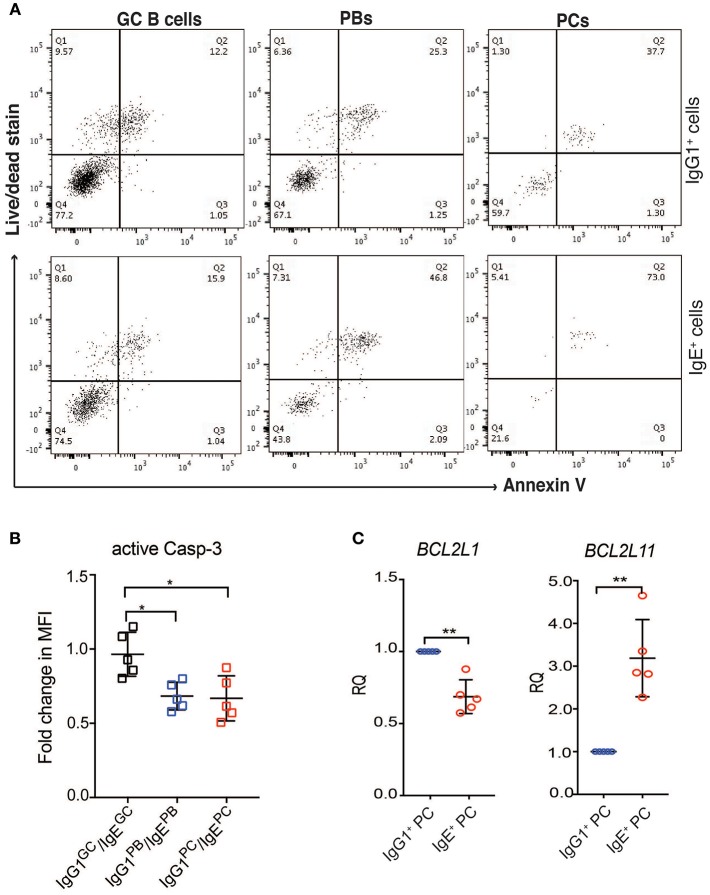
IgE^+^ PCs have increased rates of apoptosis compared to IgG1^+^ PCs. **(A)** After 24 h of reculture with IL-4 and anti-CD40, the IgE^+^ and IgG1^+^ cells were stained with annexin V and a live/dead fixable dye. The lower left quadrant within each dot plot (negative for Annexin V and live/dead stain) corresponds to the viable cells and the data shown are representative of three different experiments. **(B)** On day 10 of the culture, the activity of Caspase 3 was determined by staining with anti-active Caspase 3 antibody. The data show the fold change in MFI of active Caspase 3 within each IgG1^+^ cell population made relative to their respective IgE^+^ cell counterparts. Statistical analysis was performed using the one way ANNOVA test with Bonferroni correction (^*^P < 0.05). **(C)** RT-PCR validation of *BCL2L11* and *BCL2L1* expression in IgE^+^ and IgG1^+^ PCs. Data represent the mean +/– SD of the relative quantification (RQ). Statistical analysis was performed using the unpaired *t*-test with Welch's correction (^*^P < 0.05, ^**^P < 0.01).

Contrary to recent reports suggesting that IgE^+^ GC B cells undergo increased apoptosis compared to IgG1^+^ GC B cells (5, 6), the expression of apoptosis-associated genes in IgG1^+^ and IgE^+^ GC B cells is similar (Figure 5B). The exceptions are the pro-apoptotic regulators *BNIP3, BNIP3L*, and *HKR*, which are increased in IgG1^+^ GC cells, and *DAPK2*, increased in IgE^+^ GC cells. However, Annexin V and dead cell staining of the cells after 24 h of culture, reveals that IgE^+^ and IgG1^+^ GC B cells have similar rates of apoptosis (Figure 6A). This is also supported by their similar levels of activated caspase-3 at day 10 of the culture with IL-4 and anti-CD40 (Figure 6B), suggesting that unlike in the mouse system these cells undergo apoptosis at a similar rate. In contrast, despite increased levels of *TNFRSF13B* (TACI) and *TNFRSF17* (BCMA)*, two* important contributors of PC survival (32, 33), in IgE^+^ PBs and PCs (Supplementary Table 3), their rates of apoptosis and their expression levels of active caspase-3 are increased compared to their IgG1^+^ cell counterparts (Figures 6A,B). We find that the expression of a number of apoptosis-associated genes was either up-regulated (e.g., *BNIP2, CASP*3, *FADD*, and MAP3K5) or down-regulated (e.g., *DAPK2, BNIP3, BNIP3L, BCL2L1*, and *CASP1*) in both IgE^+^ PBs and PCs compared to their IgG1^+^ cell counterparts (Figure 5B). In addition, *BAG1, TP53INP1*, and *TP73* were down-regulated and *BCL2L11, CASP10*, and *TNFRSF25* were up-regulated only in IgE^+^ PCs (Figure 5B). The differential expression of *BCL2L1* and *BCL2L11*, which encode two well-characterized regulators of apoptosis, Bcl-xL, and Bim, respectively, in IgE^+^ and IgG1^+^ PCs was also confirmed by RT-PCR (Figure 6C).

Overall the data suggest that the apoptotic potential of IgE^+^ cells increases as they differentiate into PCs and that IgE^+^ PCs may be inhibited from exiting the cell cycle, a process that is required for the completion of the PC differentiation program (21, 22, 34).

## Discussion

A notable feature of IgE^+^ B cell development is the predisposition of IgE^+^ GC B cells to differentiate into PCs (6, 7, 9). In this study, we sought to obtain a better understanding of the IgE^+^ PC differentiation process by analyzing gene expression in human B cells at discrete stages of PC differentiation. We also compared IgE^+^ and IgG1^+^ B cells to discover isotype-specific patterns.

We identified distinct gene expression patterns at different stages of B cell differentiation into PCs and found that at each stage both IgE^+^ and IgG1^+^ cells have distinct molecular signatures with well-characterized genes of B cell function and differentiation as well as other genes of unknown function. The analysis of genes recognized as critical for either the GC reaction or PC differentiation and function confirmed the phenotype of our previously characterized IgE^+^ and IgG1^+^ cells (7).

A previous study reported that the vast majority of mouse IgE^+^ GC B cells undergo apoptosis, owing to low mIgE expression and the resulting weak BCR signaling (6). Thus, the canonical B cell differentiation programme is not observed. It was proposed that IgE BCR directly promotes the apoptosis of IgE^+^ B cells (5, 35). However, the evidence for this is conflicting, and our results are more consistent with another study in the mouse, which also demonstrated similar rates of apoptosis in IgE^+^ and IgG1^+^ GC B cells (10). Shedding further light on this matter, recent work has revealed that the expression of the ε heavy chain itself on GC B cells leads to PC differentiation uncoupled from antigen activity (5, 10). This antigen-independent PC differentiation mediated by the IgE BCR involved IRF4. The increased levels of IRF4 expression in our *in vitro* generated IgE^+^ GC-like B cells may also account, in part, for the accelerated PC differentiation of human IgE^+^ B cells. Using the curated knowledge database in IPA, we have identified two other transcriptional regulators, EGR1 and EGR2, that may contribute to this process. EGR1 has been reported to regulate PC differentiation of B cells (36) and EGR2 to be associated with T cell differentiation (37, 38). In future studies it would be interesting to determine the mechanisms by which the different expression levels of these transcription factors affect the differentiation rates of IgE^+^ and IgG1^+^ B cells.

A novel finding of our study is that as IgE^+^ and IgG1^+^ B cells differentiate into PCs their transcriptional profiles diverge, with IgE^+^ and IgG1^+^ PCs showing the greatest difference. Consistent with our previously reported results (7), we observed that a number of genes involved in the regulation of the cell cycle are differentially expressed in IgE^+^ cells. For example, the protein product of *RB1*, which is repressed in IgE^+^ PBs and PCs, can block the S-phase entry and growth by binding to the E2F1 transcription factors and inhibiting its activity (29). Similarly, *GADD45A*, which can arrest the cell cycle at the G2-M checkpoint by suppressing the CDC2/Cyclin B kinase activity (30, 31), is also down-regulated in IgE^+^ PBs and PCs. In contrast, *CDC25B* and *MYC*, two positive regulators of the cell cycle and proliferation (39, 40), are both expressed at elevated levels in the IgE^+^ cells.

In addition, using a WGCNA approach, we identified a large number of ribosomal proteins enriched in IgE^+^ PCs. It is known that the rate of translation is finely tuned to match cell proliferation (41, 42), and therefore increased ribosomal protein expression in the IgE^+^ PCs, compared to IgG1^+^ PCs, may be a consequence (or driver) of increased proliferation in these cells. Together these differences (and their downstream effects) may account for the maintenance of proliferative capacity as the IgE^+^ B cells differentiate into PCs.

Intriguingly, the analysis of the gene expression data revealed that the transcriptional profile of IgE^+^ PCs was more closely related to that of IgE^+^ and IgG1^+^ PBs than to IgG1^+^ PCs. It might be that the failure of IgE^+^ PCs to fully exit the cell cycle hinders their completion of the PC differentiation programme. Additionally, discrepancies between the IgE^+^ and IgG1^+^ PC transcriptional profiles might also be due to the up-regulation of the human mIgE_S_ (7) on becoming PCs, which distinguishes these cells from non-IgE^+^ PCs that down-regulate their mIg receptors as they become more dedicated to antibody secretion.

As seen in the mouse (10), the increased rates of apoptosis suggests that the IgE^+^ PCs generated in our tonsil B cell cultures may be short-lived PCs, which could account for some of the transcriptional differences between IgE^+^ and IgG1^+^ PCs. Similarly, a recent study, published during the review of our manuscript, reaffirmed the immature transcriptional program and relatively poor survival capacity of differentiated IgE^+^ cells isolated from the blood of peanut allergic patients (43). In support of this, our data show that IgE^+^ PCs down-regulate *BCL2L1* (Bcl-xL), which prevents apoptosis during the PC differentiation by sequestring Bim (44), a pro-apototic protein encoded by *BCL2L11* (45, 46), which is up-regulated in IgE^+^ PCs. Other pro-apoptotic associated genes that are up-regulated in IgE^+^ PCs, and which could account for their higher rates of apoptosis, include *FADD* (fas associated death domain) and *MAP3K5* (47–49). However, despite their increased rates of apoptosis, IgE^+^ PCs were expressing significantely higher levels of *TNFRSF13B* and *TNFRSF17*, which encode two very important regulators of PC survival, the transmembrane activator and CAML interactor (TACI) and the B cell maturation antigen (BCMA), respectively (32, 33, 50, 51). The differential expressions of pro- and anti-apoptotic associated genes suggests that IgE^+^ and IgG1^+^ PCs may have different survival requirements, possibly related to the microenvironment in which they reside (52, 53). This is highlighted by the serum IgE titres and the IgE-mediated responses after immunosuppressive treatments that do not affect the long-lived PCs (54–57), demonstrating the presence of long-lived IgE^+^ PCs. Further work is needed to test the predicted effects of the cell cycling and apoptosis-associated genes on IgE^+^ PC differentiation and survival.

In summary, we have defined the molecular signature of the human IgE^+^ and IgG1^+^ cell differentiation into PCs. We show that the transcriptional profile of IgE^+^ and IgG1^+^ cells diverges as these cells differentiate into PCs. At the GC stage of development, we observe similar rates of apoptosis between IgE^+^ and IgG1^+^ cells. However, IgE^+^ B cells have increased levels of IRF4 and EGR1 which may predispose these cells into PC differentiation. Significantly, IgE^+^ PCs have an immature gene expression profile that is more related to IgE^+^ and IgG1^+^ PBs than to IgG1^+^ PCs. They continue cycling and exhibit increased rates of apoptosis. Overall, our data furthers our understanding of the molecular events involved in the regulation of PC differentiation of IgE^+^ B cells and the longevity of the generated IgE^+^ PCs.

## Data Availability

The datasets generated for this study can be found in NCBI Gene Expression Omnibus, GSE99948.

## Ethics Statement

Tonsils were obtained from children undergoing routine tonsillectomies as a result of tonsillitis. Full written informed consent was given by parents or legal guardians of the donors. The study was conducted at and in accordance with the recommendations of King's College London and Guy's and St Thomas's NHS Foundation Trust and the protocol was approved by the London Bridge Research Ethics Committee (REC number 08/H0804/94).

## Author Contributions

FR and DF designed and performed experiments, analyzed data, and wrote the paper. HB performed experiments and analyzed data. HG designed experiments, analyzed data, and wrote the paper. All authors reviewed the final manuscript.

### Conflict of Interest Statement

The authors declare that the research was conducted in the absence of any commercial or financial relationships that could be construed as a potential conflict of interest.

## Abbreviations

AID: Activation-induced cytidine deaminase
EMPD: Extra-membrane proximal domain
FDR: False discovery rates
GC: Germinal Center
GO: Gene ontology
GRN: Gene regulatory network
IPA: Ingenuity Pathway Analysis
mIgE_L_: Long form of membrane IgE
mIgE_S_: Short form of membrane IgE
PB: Plasmablast
PC: Plasma cell
SOM: Self-organizing map
WGCNA: Weighted gene co-expression network analysis.
